# A rapid positive influence of *S-*ketamine on the anxiety of patients in palliative care: a retrospective pilot study

**DOI:** 10.1186/s12904-019-0499-1

**Published:** 2020-01-03

**Authors:** Eduard Falk, Daniel Schlieper, Patrick van Caster, Matthias J. Lutterbeck, Jacqueline Schwartz, Joachim Cordes, Ina Grau, Peter Kienbaum, Martin Neukirchen

**Affiliations:** 1 Interdisciplinary Centre for Palliative Medicine, Medical Faculty, Heinrich Heine University Düsseldorf, Düsseldorf, Germany; 2 Present Address: Klinik für Anästhesie, Operative Intensiv- und Palliativmedizin, Städtisches Klinikum Solingen, Solingen, Germany; 3 Department of Psychiatry and Psychotherapy, Medical Faculty, Heinrich Heine University Düsseldorf, Düsseldorf, Germany; 4 Department of Psychology, University Bonn, Bonn, Germany; 5 Department of Anesthesiology, Medical Faculty, Heinrich Heine University Düsseldorf, Düsseldorf, Germany

**Keywords:** *S-*ketamine, Esketamine, Ketamine, Anxiety, Depression, Psychological distress, Palliative care, Total pain

## Abstract

**Background:**

Patients in palliative care need rapid-acting pharmacological options for psychological distress. N-methyl-D-aspartate antagonist ketamine is known to have a fast onset of anti-depressant and anxiolytic action. Its *S*-enantiomer *S-*ketamine (or esketamine) is an analgesic used as a routine treatment for refractory pain as an intravenous infusion (0.25 mg/kg over 45 min). This study investigates whether *S-*ketamine pain therapy has a positive impact on psychological distress caused by anxiety and depression in palliative care.

**Methods:**

Patient routine data from a palliative care unit of a tertiary care hospital were used in a retrospective analysis after positive ethics approval. Eight patients, who received analgesic *S-*ketamine treatment, were compared to a control group matched by gender and age. The main analysis was conducted using three-way mixed MANOVA followed by two-way mixed ANOVA. Target variables were the values for anxiety and depression in the state-trait anxiety-depression inventory STADI. The predictor variables were the time of measurement before (T1) and after (T2) *S-*ketamine application and group membership.

**Results:**

Comparison of the *S-*ketamine group (*n* = 8; 4 male, 4 female; average age 52 years) with the control group (*n* = 8; 3 male, 5 female; average age 55 years) revealed a significant multivariate effect on anxiety and depression *F*(1, 14) = 4.78; *p* = 0.046; *r* = 0.50. The univariate comparisons showed a significant reduction of the anxiety scores from T1 to T2 in the *S-*ketamine group compared to the control group *F*(1, 14) = 10.14; *p* = 0.007; *r* = 0.65. With regard to depression, there was no significant reduction from T1 to T2 in the group comparison *F*(1, 14) = 1.60; *p* = 0.23; *r* = 0.32. No long-lasting effects on pain were found.

**Conclusions:**

Our findings show that psychological distress of patients in palliative care may improve after a single administration of *S-*ketamine, which mainly alleviates anxiety in those patients. Limitations of this study arise from non-randomization, retrospective analysis and low sample size. Therefore, further prospective and ideally randomized studies are necessary.

## Background

The total pain concept is most useful in palliative care as pain may occur on a physical, psychological, social and spiritual level [1, 2]. It is well established that physical pain and psychological distress are connected [3]. About two thirds of patients with advanced cancer suffer from pain, and more than half of those experience moderate to severe pain [4]. The WHO cancer pain relief guidelines are the standard therapy for pain [5, 6], which may achieve acceptable pain relief in over 50% of the treated patients [7]. Thus, there is still a sizeable group of patients with pain refractory to pharmacological treatment [8]. The N-methyl-D-aspartate (NMDA) receptor is an important structure in the conduction of pain signals [9] and is relevant to pathological pain states [10, 11].

Ketamine is a non-competitive NMDA-receptor antagonist that is effective in the treatment of refractory cancer pain [12–16]. Reviewing the existing data for ketamine as an adjuvant to opioids for cancer pain, Bell et al. concluded that the data is not yet sufficient to evaluate the usefulness of ketamine [17–19]. Other authors came to the same conclusion but also considered ketamine as a reasonable option for refractory, severe neuropathic and chronic pain [20–22].

Ketamine has dose dependent analgesic and anesthetic properties with sympathomimetic side effects while preserving protective reflexes [23]. It belongs to the WHO essential medicines [24]. Ketamine binds at the phencyclidine binding site of the NMDA receptor and interacts with other receptor types like opioid and cholinergic receptors [25, 26]. It is used in clinical practice as a racemic mixture in a 1:1 ratio of the *R*- and *S*-enantiomer of ketamine and as pure *S-*ketamine [27]. *S-*ketamine has the potency to block the NMDA-receptor about two times stronger than *R*-ketamine [28]. The anesthetic potency of *S-*ketamine is about twice as high as racemic ketamine and three to four times higher than *R*-ketamine [29]. Ketamine has psychotomimetic side effects including perceptual distortion and cognitive disorganization [30]. Ketamine also induces hallucinations and changes in mood and body image [23, 29]. It is abused as a recreational drug and can cause addiction [31]. The abuse of ketamine is associated with urological, neuropsychiatric, hepatobiliary and gastrointestinal complications [32]. In the treatment of depressive or anxious patients, racemic ketamine (0.50 mg/kg), as an i. v. infusion lasting 40 min, has an anti-depressive and anxiolytic effect [33, 34]. This effect begins a few hours after application, peaks at 24 h and lasts about one week [35]. The effect can be maintained through repeated application of racemic ketamine [36, 37]. There is also evidence of an anti-depressant [38, 39] and anxiolytic effect of enantiomer *S*-ketamine [29]. Recent clinical studies indicate that nasal application of *S-*ketamine in combination with an oral antidepressant reduces treatment-resistant depression [40–44]. The United States Food and Drug Administration (FDA) recently approved *S-*ketamine as a nasal spray to be used in conjunction with an oral antidepressant for the therapy of treatment-resistant depression [45]. The positive psychological effect of ketamine is attributed to an induction of neuroplasticity, which reverses the negative effect of stress and depression on neural cells and synapses [46]. Key enzymes in this process are BDNF (brain derived neurotrophic factor) [47] and mTor (mechanistic target of rapamycin) [48].

The rapid effect of ketamine on stress, anxiety and depression may be of huge importance for the treatment of psychiatric conditions of patients in palliative care. A total of 29% of patients in palliative care suffer from adjustment disorder, anxiety disorders or depression [49]. Anxiety and depression are related to a lower quality of life [50]. Depression and hopelessness are associated with a desire for hastened death [51] and higher rates of suicide [52]. Additionally, research data suggests that physical and psychological symptoms are interlinked. For example, there is a positive association of depression and anxiety with pain [53] and with physical symptom burden in patients in palliative care [54]. Moreover, anxiety and depression are associated with higher mortality in cancer patients [55]. Current pharmacological therapy options include benzodiazepines for anxiety and antidepressants for depression [56]. Benzodiazepines have a fast onset of action, but also limiting side effects like sedation, confusion, loss of coordination, addiction and paradox effects [56, 57]. Antidepressants often need about six weeks to achieve remission and do not have an effect in one third of depressive patients [58]. Six weeks is a long time for palliative care patients; too long for many. Thus, there is a need for fast-acting and reliable therapy options for these patients. Existing evidence points to a positive influence of racemic ketamine on depression and anxiety of patients in palliative care [59, 60].

In our specialized palliative care unit (SPCU) we regularly use *S-*ketamine (0.25 mg/kg i. v. infusion over 45 min) as an analgesic treatment for therapy refractory pain. *S-*ketamine is favored over racemic ketamine because it has higher analgesic and anesthetic potency and it shows less psychotomimetic side effects [29]. In a retrospective pilot study, we analyzed clinical routine data. We investigated whether an analgesic therapy with *S-*ketamine has a positive impact on psychological distress caused by anxiety and depression of patients in palliative care compared to a control group. This research question is of high interest because, to our knowledge, there is a lack of data regarding the influence of *S*-ketamine on psychological distress of patients in palliative care. Additional statistical calculations are performed to address variables with potential confounding influence, i.e. pain, need for physical care, received psychological support, received specialized palliative patient treatment, duration of anti-depressant therapy and medication with benzodiazepines, opioids and antidepressants. Furthermore, hints for longer lasting analgesic and psychotomimetic effects of *S-*ketamine are examined. The main hypothesis is that compared to a control group, an analgesic *S-*ketamine infusion reduces psychological distress caused by anxiety and depression. Additionally, it is hypothesized that even after taking the confounding variables into account, the positive effect of *S-*ketamine on psychological distress remains.

## Methods

### Study design

This pilot study is a retrospective analysis of routine patient data over a one-year period (April 2016 to March 2017). Inclusion criteria for the *S-*ketamine group were a minimum age of 18 years old, analgesic treatment with a *S-*ketamine infusion and sufficient data for comparison before and after *S-*ketamine administration. Inclusion criteria for the control group were a minimum age of 18 years old and sufficient data for comparisons at two measurement points. Patients with the first measurement point on the day of admission were excluded from the control group to avoid confounding influences of admission procedures. Patients from both the *S*-ketamine group and control group were offered the same kind of specialized palliative care treatment. The only difference was that the control group did not need *S*-ketamine for pain control and therefore was not treated with *S*-ketamine. This study uses the STROBE guidelines for reporting observational studies [61].

### Setting

The analyzed data were collected in a clinical routine during a standard inpatient treatment in the SPCU of a university hospital in Germany. The SPCU offers specialized palliative care with beds for eight patients. The patients usually suffer from many different and complex symptoms. The team consists of physicians, nurses, psychologists, physiotherapists, art therapists, social services professionals, spiritual welfare professionals, volunteers and others.

### Measurements

#### Primary study outcome variables

The State Trait Anxiety Depression Inventory (STADI) [62] is a validated questionnaire for evaluating depression and anxiety as states and as traits [63]. STADI has been available since 2013 and used in clinical settings [64] including this study’s SPCU for routine assessment. The internal consistency is at least *α* = 0.81. Standard values are available based on a representative test group (*N* = 3150) [63]. The STADI allows the calculation of scores for anxiety and depression. The global score is the sum of the anxiety and the depression scores and can be interpreted as psychological distress in the sense of negative affectivity. The state and trait section of the questionnaire consists of 20 items. For depression, anxiety and the global score, standardized comparison values are available according to age and gender. Using these standard tables, the individual raw values are normalized into T-scores. A T-score is a standardized score with a mean of 50 and a standard deviation of 10. A T-value > 60 is classified as pathological [62]. The state part of this questionnaire was evaluated upon admission and at regular intervals of 1 to 5 days during the stay in the SPCU.

#### Potential confounding variables of primary outcomes

**Pain** is part of the Palliative Symptom Burden Score (PSBS) [65, 66], which is routinely used in the SPCU. Pain was assessed using the numerical rating scale for pain (NRS) with a range from no pain (0) to the worst imaginable pain (10). Pain with NRS values below 3 is considered to be mild, values between 3 and 6 are moderate and values over 6 are high. During standard care on the ward, the PSBS is assessed three times a day at intervals of 8 h; the first, second and third evaluations take place during the periods from 12 am to 8 am, from 8 am to 4 pm and from 4 pm to 12 am, respectively. There is a positive association between depression and pain [53], so the NRS of the PSBS is considered to be a potential confounder.

##### Activities and existential experiences of life (AEDL) score

The AEDL is a measurement tool based on the concept of nursing process management [67]. The following 9 aspects are rated on five-level Likert-items (range: 0–4): *resting/sleeping, moving, washing/dressing, eating/drinking, excretion, communicating, finding occupation/finding sense and meaning, safe environment* and *social surroundings*. A total scale value has a range from 0 to 36. Higher values represent a greater limitation and thus a higher need for care and support. On the ward, the AEDL is evaluated once a day. The AEDL is considered to be a confounder because stronger limitations in physical functioning, role functioning and social functioning are significantly correlated with higher anxiety and depression [50].

##### Psycho-oncological treatment

The patients received psychological and psycho-oncological support in the form of psychotherapy, art therapy and animal-assisted therapy. Psychological support can reduce anxiety and depression [56]. Thus, we used the time spent on psycho-oncological treatment as a confounding variable. Time was used to measure the dose of the interventions because psychotherapy has been shown to have a dose-effect-relationship [68, 69]. The total amount of therapy time in minutes, up to the considered points of measurement, was used to indicate the amount of psychological and psycho-oncological treatment.

##### Palliative care treatment

Patients received a specialized palliative treatment every day of the stay through the synergistic work of all employed professionals. The number of days on the SPCU was used as a measure of the extent of this specialized, inpatient palliative care. Because the synergistic work of all employed professionals including physicians and psychologists may reduce anxiety and depression, the amount of palliative care treatment is considered to be a confounding variable.

##### Days with antidepressants

Because the positive effect of antidepressants on mood depends on the length of intake, the number of days with antidepressants on the SPCU is considered to be a confounder.

##### Medication

Any intake of antidepressants, benzodiazepines or opioids on the points of measurement was considered, regardless of the time of day or whether it was the standard medication or was given on demand. Because the intake of antidepressants, benzodiazepines and opioids may exert an acute effect on mood, these medications are considered to be confounders.

#### Secondary study outcome variables

To assess whether there is a prolonged positive influence of *S*-ketamine on pain, the NRS of the PSBS was considered as a secondary variable. Restlessness and anxiety were considered as possible psychotomimetic side effects of *S-*ketamine. The combined item restlessness/anxiety is also part of the PSBS which is routinely used in the SPCU. The ordinal scaled item has a range from 0 to 4 with 0 for *no impairment*; 1 for *occasionally impaired - patient can express the cause for restlessness/anxiety*; 2 for *restlessness/anxiety are occurring frequently - care needed*; 3 for *restlessness/anxiety are occurring despite medication,* and 4 for *pronounced restlessness, panic and/or suicidal tendencies*.

### Time of assessments

Because STADI was assessed several times a week (but not daily), the time span between STADI measurements was up to 4 days. For the patients requiring *S-*ketamine treatment, the first measurement point (T1) was the last time the STADI was evaluated before *S-*ketamine administration. The second measurement point (T2) was the first STADI evaluation after *S-*ketamine administration. In the control group, T1 was the first STADI evaluation from the second day of the stay, and T2 was the time of the next STADI evaluation. Furthermore, for some analyses, the morning before *S-*ketamine administration (Z1) and the morning after *S-*ketamine administration (Z2) were taken into account. For the control group, Z1 was the morning of T1 and Z2 was the morning of the day after T1. The scores of the first of the three daily evaluations were used for the main analyses using the variables pain and restlessness/anxiety of the PSBS.

### Statistical analyses

Data were analyzed using IBM SPSS 25.0 for Macintosh [70]. After recoding negative items of STADI, reliability was calculated for STADI and for AEDL to T1 and T2 using Cronbach’s α. Test-retest-reliability was calculated between the scores of the first and second daily evaluation at T2 with Pearson’s *r* for pain and Spearman’s ρ for restlessness/anxiety. A propensity score was calculated using logistic regression with group membership (*S*-ketamine, control) as the target variable and age and gender as predictor variables. Patients of the control group were matched 1:1 without replacement to the *S-*ketamine group using the nearest neighbor method without a specified caliper width. To measure the balance between the groups, *z*-differences were calculated [71]. Absolute z-differences lower than 1.41 are considered as appropriate [72]. The main analyses were conducted using multivariate and univariate analyses of variance. Target variables were STADI T-values for anxiety and depression as well as psychological distress, i.e. the combination of anxiety and depression. The predictors used for the analyses were: 1) The group membership (group; *S-*ketamine vs. control; between subject factor), 2) The measurement points (time; T1 vs. T2; within subject factor) and 3) The difference between STADI anxiety and depression values (here abbreviated as *anxdep*; anxiety vs. depression; within subject factor). Subsequently, interval scaled confounders were included separately as covariates in an analysis of covariance. Nominal scaled confounders (medication) were included separately as predictors in a multivariate analysis of variance. To determine if there is a difference of medication intake between T1 and T2, thus requiring a separate analysis of both points of measurement in the multivariate analyses of variance, the association of medication intake at T1 and T2 was calculated with the phi (φ) coefficient. In order to analyze the prolonged effect of *S-*ketamine on pain, a univariate analysis of variance was calculated, with pain as a dependent variable, and group membership (group; *S-*ketamine vs. control; between subject factor) and measurement points (time; Z1 vs. Z2; within subject factor) as predictors. A Wilcoxon signed-rank test was used for comparisons of repeated measures of restlessness/anxiety at Z1 and Z2 as a measure for prolonged psychotomimetic side effects of *S-*ketamine. Assumptions of normal distribution, homogeneity of variance and homogeneity of covariance matrices were tested before analyzing the data via analyses of variance.

Because of the exploratory approach of this study, different hypotheses were tested, while no adjustments for multiple comparisons were made to correct for the familywise error. Considered as significant results for all statistical analyses were *p* < 0.05. Results with 0.05 < *p* < 0.10 were regarded as a trend to significance. Effect size *r* for statistical comparisons were calculated with the following equations [73]:

$$ r=\sqrt{\frac{F\;\left(1,d{f}_{Residual}\right)}{F\;\left(1,d{f}_{Residual}\right)+d{f}_{Residual}}} $$

and

$$ r=\frac{z}{\sqrt{N}} $$

According to Cohen, values of *r* of 0.1, 0.3 and 0.5 were classified as small, medium and large effect sizes, respectively [74].

## Results

### Sample description

**Fig. 1:**
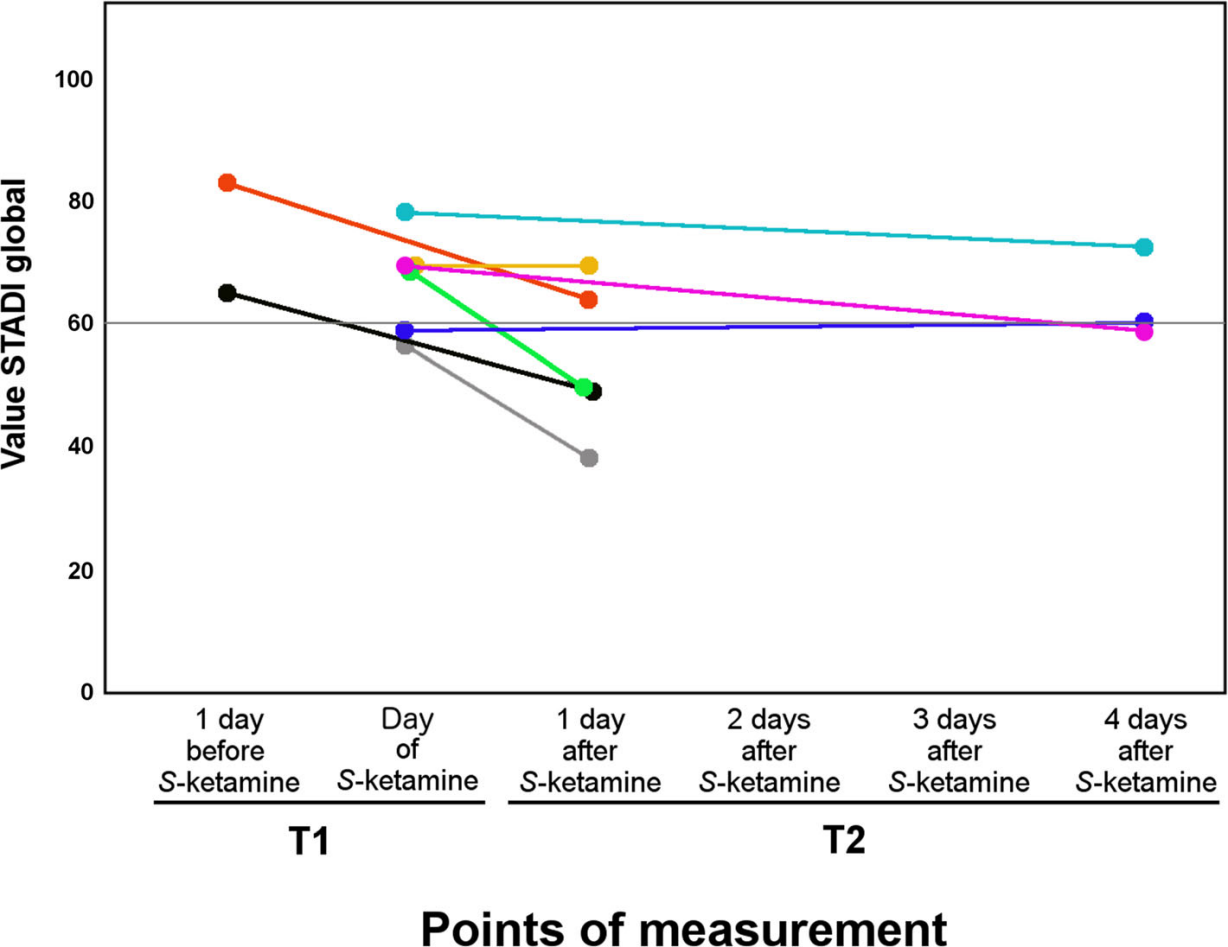
Changes in the STADI global values of the S-ketamine group from T1 to T2. STADI values were assessed on several days a week and therefore not available 2 to 3 days after S-ketamine application. The circles at the beginning and end of each line are measured data points, while the connecting lines are shown to identify each individual

There were *n* = 8 patients who were treated with *S-*ketamine for refractory pain with sufficient data available. In the control group, seventeen patients with sufficient data at two points in time were included. Two patients with the first STADI evaluation on the day of admission were excluded to prevent the admission procedure from confounding the data. Accordingly, eight patients from the *S-*ketamine group and fifteen patients from the potential control group contribute to the final analyses. The control group was adjusted to the *S*-ketamine group by age and gender, using propensity score matching. The absolute *z*-difference for age (0.38) and gender (female: 0.51) were below 1.41 and thus appropriate. The sample characteristics are shown in Table 1. Figure 1 shows the changes of the STADI global values from T1 to T2 for the *S-*ketamine group. As can be seen from the trajectories, 5 out of 8 patients improved from a clinical point of view, i.e. their STADI global levels decreased by more than 10 points (Fig. 1).

**Table 1:** Sample characteristics of the *S-*ketamine and the control group

Variables	Categories	*S-*ketamine group	Control group
Group size ^a^		8	8
Gender ^a^	Female	4	5
	Male	4	3
Age ^b^		52.13 ± 13.25	54.63 ± 13.23
Diagnosis at admission ^a^	Breast cancer	0	3
	Cancer of unknown primary	1	1
	Cervical cancer	1	1
	Glioblastoma	1	0
	Colorectal cancer	1	0
	HIV	1	0
	Liver cancer	0	1
	Lung cancer	2	0
	Ovarian cancer	0	1
	Pancreatic cancer	0	1
	Prostate cancer	1	0
Length of stay in SPCU (days) ^b^		14.63 ± 7.69	13.00 ± 3.42
Mode of discharge ^a^	Home	0	4
	Hospice	3	1
	Other clinic	0	2
	Died on the ward	5	1
Points of measurement (days) ^b^	T1	5.63 ± 2.88	3.38 ± 1.06
	T2	8.00 ± 3.70	7.50 ± 2.45
	Z1	5.88 ± 2.90	3.38 ± 1.06
	Z2	6.88 ± 2.90	4.38 ± 1.06
STADI anxiety ^b^	T1	68.88 ± 11.01	57.38 ± 13.38
	T2	55.63 ± 11.73	57.50 ± 12.46
STADI depression ^b^	T1	66.38 ± 10.88	59.25 ± 12.51
	T2	57.75 ± 12.75	59.00 ± 13.40
STADI global ^b^	T1	68.38 ± 8.80	59.38 ± 13.66
	T2	57.38 ± 11.87	59.00 ± 13.27
Pain ^b^	Z1	3.88 ± 1.64	2.88 ± 2.10
	Z2	3.50 ± 1.77	2.75 ± 1.67
Restlessness/anxiety ^c^	Z1	1.00 (1.00–1.75)	1.00 (0–1.75)
	Z2	1.00 (1.00–1.00)	1.00 (0–1.75)

^a^= *n*

^b^= *M* ± *SD*

^c^= *Mdn* (*IQR*)

Descriptively, the *S-*ketamine group differs from the control group in terms of parts of the sample characteristics. More patients from the *S-*ketamine group than patients from the control group died on the ward, indicating that the control group was fitter than the *S*-ketamine group. Furthermore, at T1 the *S-*ketamine group had higher STADI values (STADI > 60) compared to the control group (STADI < 60). The *S-*ketamine group had moderate pain (NRS ≥ 3), compared to the control group, which had mild pain (NRS < 3) at Z1 and Z2. In Fig. 1, the changes of the values of the STADI global scores are more pronounced if T2 is one day after *S-*ketamine treatment rather than 4 days.

The confounding variables: pain, AEDL, psycho-oncological treatment, days with antidepressants, palliative care treatment and medication are shown in Table 2. Moreover, with regard to the confounding variables at T1 and T2, the *S-*ketamine group differs descriptively from the control group. The *S-*ketamine group had moderate pain and the control group had mild pain at both points of measurement. In relation to the AEDL, which represents need for care, the *S-*ketamine group had higher scores at both measurement points than the control group. The *S-*ketamine group made less use of the psycho-oncological treatment than the control group at both points of measurement. With regard to medication, more patients from the *S-*ketamine group took antidepressants at T1 compared to the control group. Furthermore, all patients of the *S-*ketamine group took opioids at both measurement points.

**Table 2:** Confounding variables

Confounder	Points of measurement	*S-*ketamine group		Control group	
Pain ^a^	T1	4.00 ± 1.85		2.88 ± 2.10	
	T2	3.50 ± 1.77		2.88 ± 1.73	
AEDL ^a^	T1	14.50 ± 7.69		8.00 ± 4.63	
	T2	15.88 ± 7.70		9.38 ± 5.24	
Psycho-oncological treatment (minutes) ^a^	T1	53.75 ± 55.92		72.00 ± 60.47	
	T2	68.75 ± 68.86		124.50 ± 90.17	
Days with antidepressants ^a^	T1	2.38 ± 2.97		1.25 ± 1.83	
	T2	3.88 ± 4.12		2.88 ± 3.31	
Palliative care treatment (days) ^a^	T1	5.63 ± 2.88		3.38 ± 1.06	
	T2	8.00 ± 3.70		7.50 ± 2.45	
Benzodiazepines ^b^	T1	yes	6	yes	5
		no	2	no	3
	T2	yes	6	yes	4
		no	2	no	4
Antidepressants ^b^	T1	yes	6	yes	3
		no	2	no	5
	T2	yes	6	yes	5
		no	2	no	3
Opioids ^b^	T1	yes	8	yes	7
		no	0	no	1
	T2	yes	8	yes	7
		no	0	no	1

^a^= *M* ± *SD*

^b^= *n*

### Primary study outcome variables

**Fig. 2:**
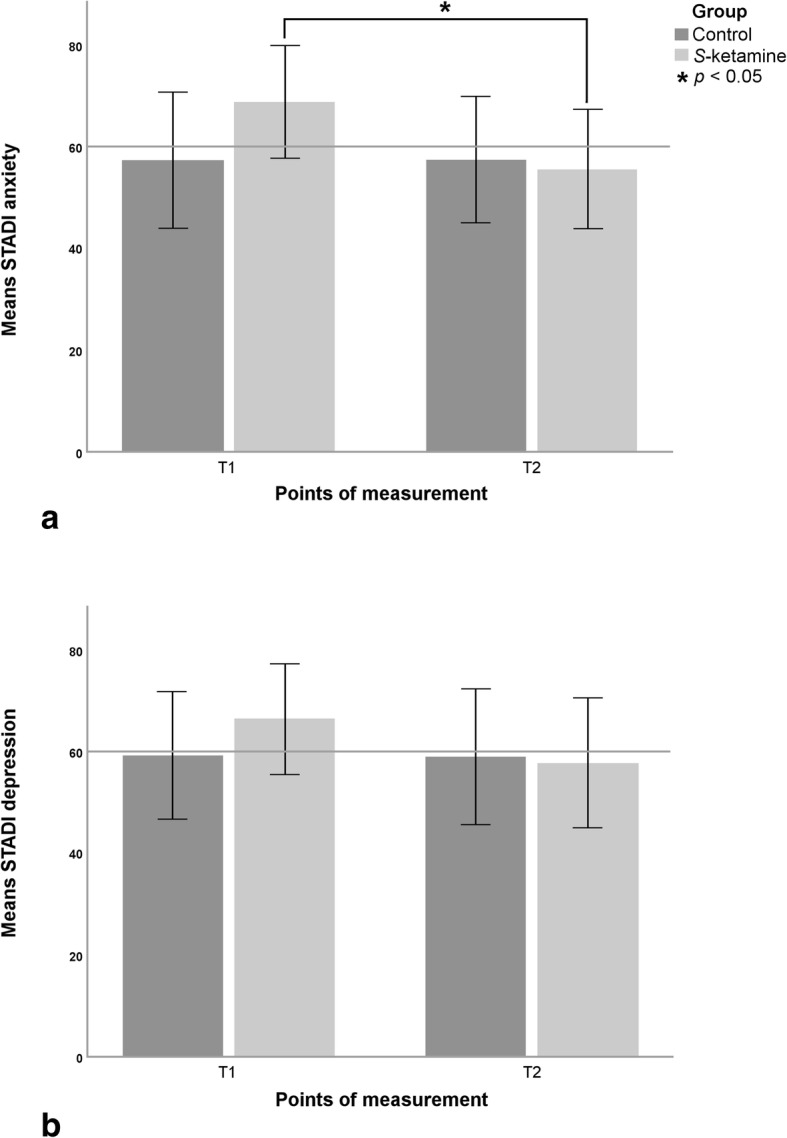
Group means ± SD for STADI anxiety (**a**) and depression (**b**) values at T1 and T2. * p: statistical significance *p* < 0.05 of mean differences

Cronbach’s α for all STADI scales at T1 and T2 was above 0.91 and therefore the reliability was classified as good. Three-way mixed multivariate analyses of variance (MANOVA) were conducted with anxiety and depression as target variables. Predictors were group, time and anxdep. The results are shown in Table 3. Figure 2 shows STADI values of anxiety and depression according to group membership and measurement points. There was a significant interaction between group and time with a medium effect size, caused by the reduction of anxiety and depression, i.e. psychological distress, from T1 to T2 in the *S-*ketamine group. There was no significant interaction between group, time and anxdep, suggesting that there was a similar effect of *S-*ketamine on anxiety and depression in the *S-*ketamine group.

**Table 3:** Three-way mixed MANOVA; target variables: anxiety, depression; predictor variables: group, time and anxdep

		Test statistics	Significance 2-tailed	Effect size
STADI scales	Effect	*F*(1, 14)	*p*	*r*
Anxiety and depression	Group	0.60	0.45	0.20
	Anxdep	0.11	0.75	0.09
	Group x anxdep	0.17	0.69	0.11
	Time	4.89	0.044*	0.51
	Group x time	4.78	0.046*	0.50
	Anxdep x time	0.76	0.40	0.23
	Group x anxdep x time	1.05	0.32	0.26

*p: statistical significance *p* < 0.05

Subsequently conducted two-way mixed analyses of variance (ANOVA) were calculated separately for anxiety and depression using group and time as predictors (Table 4). Regarding anxiety, group-by-time interaction was significant with a large effect size. Pairwise comparison of the changes of the STADI anxiety values showed a significant reduction in the *S-*ketamine group *F*(1, 14) = 19.89; *p* = 0.001; *r* = 0.77 from T1 to T2 but not in the control group *F*(1, 14) = 0.002; *p* = 0.97; *r* = 0.01 (Fig. 2A). There was no significant interaction between group and time regarding depression but there was still a medium effect size (Table 4; Fig. 2B).

**Table 4:** Two-way mixed ANOVA; target variables: anxiety and depression; predictor variables: group and time

		Test statistics	Significance 2-tailed	Effect size
STADI scale	Effect	*F*(1, 14)	*p*	*r*
Anxiety	Group	0.71	0.41	0.22
	Time	9.76	0.007*	0.64
	Group x time	10.14	0.007*	0.65
Depression	Group	0.31	0.59	0.15
	Time	1.80	0.20	0.34
	Group x time	1.60	0.23	0.32

*p: statistical significance *p* < 0.05

### Potential confounding variables of primary outcomes

Test-retest-reliability for pain at T2 showed high correlation (*r* = 0.95; *p* < 0.001; *n* = 15) and Cronbach’s α of AEDL at T1 and T2 was above 0.87 and therefore the reliability was classified as good. The confounders pain, AEDL, psycho-oncological treatment, days with antidepressants and palliative care treatment are displayed in Table 2. To consider the influence of these interval-scaled confounders, two-way mixed multivariate analyses of covariance (MANCOVA) were calculated, using anxiety and depression as target variables. The predictors were group and time. Each confounder with its value to T1 and T2 was included separately as a covariate in each analysis. The results of the two-way mixed MANCOVAs are shown in Table 5. There was a significant interaction of group and time, even after taking into account the confounding variables as covariates. In the following two-way mixed analyses of covariance (ANCOVA), there were significant group-by-time interactions for anxiety with large effect sizes. However, the same kind of ANCOVA for depression was not significant, but had small to medium effect sizes.

**Table 5:** Two-way mixed MANCOVA (multivariate) and two-way mixed ANCOVA (univariate) with confounders as covariates

		Multivariate		Univariate					
		Anxiety & depression		Anxiety			Depression		
Covariates at T1 and T2		Test statistics	Sig. 2-tailed	Test statistics	Sig. 2-tailed	Effect size	Test statistics	Sig. 2-tailed	Effect size
	Effect	*F*(2, 12)	*p*	*F*(1, 13)	*p*	*r*	*F*(1, 13)	*p*	*r*
Pain	Group	0.23	0.79	0.49	0.50	0.19	0.32	0.58	0.15
	Time	4.90	0.028*	8.79	0.011*	0.64	1.73	0.21	0.34
	Group x time	5.31	0.022*	9.15	0.010*	0.64	1.54	0.24	0.33
AEDL	Group	0.21	0.81	0.09	0.77	0.08	0.08	0.78	0.08
	Time	3.44	**0.066**^**+**^	7.20	0.019*	0.60	2.61	0.13	0.41
	Group x time	5.32	0.022*	9.48	0.009*	0.65	1.60	0.23	0.33
Psychooncological treatment (minutes)	Group	0.67	0.53	1.45	0.25	0.32	0.48	0.50	0.19
	Time	7.77	0.007*	16.76	0.001*	0.75	4.60	**0.051**^**+**^	0.51
	Group x time	4.27	0.040*	8.22	0.013*	0.62	0.68	0.43	0.22
Days with antidepressants	Group	0.10	0.91	0.22	0.65	0.13	0.10	0.76	0.09
	Time	2.43	0.13	4.48	**0.054**^**+**^	0.51	0.61	0.45	0.21
	Group x time	4.95	0.027*	9.39	0.009*	0.65	1.51	0.24	0.32
Palliative care treatment (days)	Group	0.15	0.86	0.32	0.58	0.15	0.18	0.68	0.12
	Time	2.66	0.11	4.71	0.049*	0.52	4.95	0.044*	0.53
	Group x time	4.41	0.037*	6.89	0.021*	0.59	0.33	0.58	0.16

*p: statistical significance *p* < 0.05

^+^*p*: trend to statistical significance: 0.05 < *p* < 0.10

The intake of benzodiazepines, antidepressants and opioids is shown in Table 2 according to group and points of measurement. The intake of these medications was considered separately for confounding influences by including these nominal-scaled variables as predictors in the analyses. The analyses were conducted with a three-way mixed MANOVA with anxiety and depression as target variables. The predictors were group, time and medication. The intake of opioids could not be included in these analyses because there were no patients in the *S-*ketamine group who did not take opioids.

To assess whether there is a difference of medication intake between the points of measurement, phi (φ) was calculated as a measurement of association of medication intake between T1 and T2. There was a strong and significant relationship between the intake of antidepressants with φ(16) = 0.76; *p* = 0.002 and benzodiazepines with φ(16) = 0.59; *p* = 0.018 on T1 and T2. Because the intake of medication is relatively equivalent at T1 and T2, it did not matter which point of measurement was chosen in the further analyses. Therefore, the intake of benzodiazepines and antidepressants on T1 was used as a between-subject variable in a three-way mixed MANOVA. Target variables were anxiety and depression, and predictor variables were group, time and medication. The results are shown in Table 6. Even after considering the intake of benzodiazepines at T1 as a confounding variable in the three-way mixed MANOVA, there was a significant group-by-time interaction. Furthermore, when controlling for antidepressants there was a tendency for a significant group-by-time interaction. There was no significant interaction between group, time and medication in these analyses, suggesting no discernible association between the intake of benzodiazepines or antidepressants and the improvement of the *S-*ketamine group.

**Table 6:** Three-way mixed MANOVA (multivariate) and three-way mixed ANOVA (univariate) analysis of the effect of medication

		Multivariate		Univariate					
		Anxiety & depression		Anxiety			Depression		
		Test statistics	Sig. 2-tailed	Test statistics	Sig. 2-tailed	Effect size	Test statistics	Sig. 2-tailed	Effect size
Med.	Effect	*F(*2, 11)	*p*	*F(*1, 12)	*p*	*r*	*F*(1, 12)	*p*	*r*
B T1	Group	0.59	0.57	0.92	0.36	0.27	0.05	0.83	0.06
	Time	3.23	**0.079**^**+**^	5.96	0.031*	0.58	0.95	0.35	0.27
	B T1	0.94	0.42	0.19	0.68	0.12	1.65	0.22	0.35
	Group x B T1	0.87	0.45	0.54	0.48	0.21	0.11	0.74	0.10
	Time x B T1	0.18	0.84	0.33	0.58	0.16	0.04	0.84	0.06
	Group x time	4.28	0.042*	6.71	0.024*	0.60	0.44	0.52	0.19
	Group x time x B T1	0.99	0.40	0.03	0.86	0.05	1.35	0.27	0.32
A T1	Group	0.01	0.99	0.01	0.91	0.03	0.02	0.90	0.04
	Time	2.95	**0.094**^**+**^	6.24	0.028*	0.58	2.14	0.17	0.39
	A T1	3.17	**0.082**^**+**^	6.80	0.023*	0.60	1.53	0.24	0.34
	Group x A T1	0.70	0.52	1.52	0.24	0.34	0.41	0.54	0.18
	Time x A T1	0.57	0.58	1.21	0.29	0.30	0.46	0.51	0.19
	Group x time	3.03	**0.090**^**+**^	5.62	0.035*	0.56	0.99	0.34	0.28
	Group x time x A T1	1.41	0.29	0.15	0.71	0.11	0.86	0.37	0.26

Med.: Medication

B T1: Benzodiazepines at T1

A T1: Antidepressants at T1

*p: statistical significance *p* < 0.05

^+^*p*: trend to statistical significance: 0.05 < *p* < 0.10

Three-way mixed ANOVAs were calculated for benzodiazepines and for antidepressants. The effect of antidepressants was analyzed with the three-way mixed ANOVA despite the related non-significant group-by-time interaction in the three-way mixed MANOVA. The results are shown in Table 6. These analyses showed significant group-by-time interactions for anxiety with a large effect size. There was also a significant effect of antidepressants intake on T1. This effect is caused because the overall anxiety scores of patients who took antidepressants on T1 (*M* = 65.89; *SD* = 10.39) were higher than those patients who did not take antidepressants on T1 (*M* = 52.07; *SD* = 7.16). For the target variable depression there were no significant group-by-time interactions. These analyses showed small effect sizes. There was no significant interaction between group, time and medication for either of the target variables.

### Secondary study outcome variables

Test-retest-reliability for restlessness/anxiety at T2 showed high correlation (ρ = 0.92; *p* < 0.001; *n* = 15) and was therefore classified as good. The item restlessness/anxiety was considered to be a measure of persistent psychotomimetic side effects of *S*-ketamine. Possible changes in restlessness/anxiety, from the morning before *S-*ketamine administration to the morning after, were analyzed with the Wilcoxon Signed Rank Test. The predictor variables were the measurement points Z1 and Z2. There were no significant changes in restlessness/anxiety *T* = 0; z = − 1.00; *p* = 0.32; *r* = − 0.35 from Z1 to Z2. Thus, we found no evidence for a persistent psychotomimetic effect of *S-*ketamine.

The change in pain from Z1 to Z2 was analyzed between the groups using a two-way mixed ANOVA. The predictor variables were time and group. There was no significant interaction between group and time *F* (1, 14) = 0.11; *p* = 0.75; *r* = 0.09. This means that regarding pain, the changes in the *S-*ketamine group from Z1 to Z2 were not significantly different from those of the control group of Z1 to Z2. Thus, we found no evidence of a prolonged analgesic effect of *S-*ketamine.

What influence does the psychological distress at T1 have on the results? Descriptively, the *S*-ketamine and the control group differed according to the STADI values of anxiety and depression at T1. To adjust these STADI scale values, and thus make the groups more comparable, an alternative propensity score matching strategy was used, i.e. with STADI global score at T1, together with age and gender (see Additional files). Using this alternative matching strategy, we had STADI values of over 60 at T1 as well as for the control group (Additional file 1: Table S1; Additional file 7: Figure S1). Even with this matching, a significant effect of *S-*ketamine on anxiety remained in the ANOVA and ANCOVA (Additional file 2: Table S2, Additional file 3: Table S3, Additional file 4: Table S4, Additional file 5: Table S5, Additional file 6: Table S6 and Additional file 7: Figure S1a).

## Discussion

This retrospective pilot study provides the first evidence of a positive effect of *S-*ketamine on the psychological distress of patients in palliative care. We find a multivariate effect on depression and anxiety with a primary effect on anxiety. Our result corresponds to earlier studies showing that ketamine racemate shows similar effects in patients in palliative care [59, 60, 75]. To our knowledge, the effect of the purified enantiomer *S*-ketamine on patients in palliative care has not been analysed previously. *S-*ketamine is reported to have a positive effect on anxiety in surgical patients without palliative diagnosis [29], and has recently been approved as nasal spray by the FDA – but only when used in conjunction with an oral antidepressant and only for the therapy of treatment-resistant depression [45]. We hope that for patients with a life-limiting disease, *S*-ketamine can be useful outside the FDA approval. This study may be a first step towards the approval to treat anxiety of patients in palliative care with *S*-ketamine.

The positive effect of *S-*ketamine was mainly on anxiety with no significant effect on depression. The influence of *S-*ketamine on anxiety had consistently large effect sizes. Our data indicate that S-ketamine treatment may be effective in routine clinical practice. In our study, *S*-ketamine reduced the global STADI values by a clinically relevant level in 5 out of 8 patients (Fig. 1). Thus, we estimate the number needed to treat is approximately 2. Further studies are needed to establish the effectiveness.

The influence of *S-*ketamine on depression showed mainly medium effect sizes. The significant effect with a large effect size of *S-*ketamine on psychological distress was mainly caused by the reduction in anxiety. However, the analyses also showed that the changes in anxiety and depression due to *S-*ketamine were similar. Thus, *S-*ketamine had an analogical effect on anxiety and depression. Even after taking the confounding variables into account, the significant effect on anxiety remained. There was also no evidence of persistent psychotomimetic side effects in the *S-*ketamine group until the next morning. Furthermore, there were no indications of a sustained pain reduction by *S-*ketamine until the next morning in the group comparison.

The pronounced effect of *S-*ketamine on the anxiety of patients in palliative care may be related to the peculiarities of this group of patients. In a case report on two hospice patients receiving a single dose of ketamine racemate (0.50 mg/kg bolus per os) to treat psychological distress, there was a positive effect on anxiety and depression, with a more pronounced reduction in anxiety over the first four days [60]. In addition, both patients experienced an improvement in pain perception with a maximum of four and eight days after ketamine administration. In a feasibility study, the effect of daily oral administrations over 28 days of ketamine racemate (0.50 mg/kg bolus per os) on anxiety and depression was investigated [75]. There was a significant response (reduction of questionnaire scores > 30%) of anxiety to ketamine racemate after three days with a medium effect size (*d* = 0.67). For depression, there was a significant response after 14 days with a large effect size (*d* = 1.14). After 28 days a significant effect was sustained with large effect sizes for anxiety (*d* = 1.34) and depression (*d* = 1.34). However, pain was unchanged [75].

The results of our work and the two hospice studies suggest that *S-*ketamine and ketamine racemate act primarily on anxiety in patients with a life-limiting disease. Whether this is a special pattern of action in these group of patients requires further clarification.

In our study, a positive effect of *S-*ketamine on depression could not be identified in the group comparisons. However, the initial univariate group comparison (Table 4) found a medium effect size (*r* = 0.32) for depression. A post-hoc sample size calculation with G*Power 3.1 [76, 77] showed that a total of *n* = 20 patients would be necessary to determine a significant effect on depression for a group-by-time interaction in a two-way mixed ANOVA. Thus, according to our data, a prospective study would need 20 patients or more.

In this study, the descriptive interpretation of the data suggests that more patients in the *S-*ketamine group than in the control group died on the ward (Table 1). A causal relationship to *S-*ketamine is not plausible for the following reasons: In general, about 60% of the inpatients in the SPCU die on the ward [78]. Thus, the mortality of the *S-*ketamine group can be considered average. Furthermore, a study by Irwin et al. [75] showed that daily oral administrations of ketamine racemate for 28 days led to no serious adverse events. There were no changes in vital signs (blood pressure, heart rate and respiratory rate) during the course of their study. A mild increase of symptoms in 12.5% of patients were related to diarrhea, sleeping problems and restlessness. In addition, the patients showed a decrease in symptom burden related to gastrointestinal, neurological and psychiatric symptoms. Further studies on the effect of ketamine racemate on the mental health of hospice patients [59, 60] and of psychiatric patients [79] also report a low rate of adverse events. The most frequent adverse events in patients receiving ketamine racemate (0.50 mg/kg over 40 min i. v.) as a therapy of treatment resistant depression were drowsiness, dizziness, poor coordination and a strange or unreal feeling [79]. These symptoms were mostly experienced in the first two hours after the beginning of the infusion, diminishing after four hours and practically ceasing after 24 h.

A descriptive synopsis of the data collected in this study suggests that the *S-*ketamine group was a group of patients with a higher symptom burden than the control group. The *S-*ketamine group showed, at T1, STADI T-values over the critical limit of 60. In addition, the *S-*ketamine group reported moderate pain at both time points. Furthermore, the *S-*ketamine group showed, at both points of measurement, a need for more care than the control group (as indicated by the AEDL score). On average, the *S-*ketamine group also had less psycho-oncological treatment. It is plausible that the reduced physical status of the *S-*ketamine group, which was manifested in increased need for care and increased mortality on the ward, reduced the possibility of participating in psycho-oncological interventions.

### Limitations

The limitations of this study results from its retrospective design, which prevented randomization. Because of the retrospective design, the data is not optimal to measure the effect of *S-*ketamine. The best interval to measure the maximum effects of ketamine or *S-*ketamine is one day after administration of ketamine or *S-*ketamine. In our study, there were several days between T1 and T2 in the *S-*ketamine group (Fig. 1), which may have reduced the measured effect of *S-*ketamine on anxiety and depression. However, the obtained data (Fig. 1) showed a stronger reduction of psychological distress caused by anxiety and depression one day after *S-*ketamine administration than four days after *S-*ketamine administration. Thus, these data are consistent with the time course of the effect of ketamine [80]. Furthermore, the retrospective approach does not allow an evaluation of how the patients have perceived the effects of *S-*ketamine and how they assess the benefits and risks related to *S-*ketamine treatment. Non-randomization can lead to systematic bias. In this study, the group membership was systematic, because only the patients suffering from refractory pain received *S-*ketamine. In this context, additional patient data indicates that the *S-*ketamine group was a patient population with a higher symptom burden. Thus, the patients in the *S-*ketamine group, who suffered from refractory pain, still had other physical and psychological symptoms, which distinguished them from patients in the control group. The STADI scores for anxiety and depression were significantly higher in the *S-*ketamine group than in the control group at T1. To minimize statistical errors arising from the selection of the control group, we generated another control group. This alternative matching strategy takes psychological distress into account. Still, when using the alternative matching strategy, our results on the effect of *S-*ketamine are essentially the same (see Additional files). To avoid other statistical errors, we calculated Cronbach’s alpha and test-retest-reliability to ensure good reliability of our instruments. For the analyses of variance, we ensured that all assumptions of normal distribution, homogeneity of variance and homogeneity of covariance matrices were met.

A further limitation of our study is the non-randomization and the small sample size, which makes it difficult to generalize the data. Despite this limitation, our results provide the basis for prospective studies, which will be needed as soon as *S*-ketamine is approved as nasal spray by the European Medicines Agency and other regulators around the world. Subsequent studies will provide an empirical basis for the treatment of anxiety and depression with *S-*ketamine of patients in palliative care. The first step would be prospective feasibility studies, including qualitative data if sample size is expected to be low. Further studies could include double-blinded, randomized and placebo-controlled trials. During the course of these studies, the questions on the pattern of effects, the optimal forms of application and the choice of medication *S*-enantiomer vs. racemate should be considered.

## Conclusions

The results of this retrospective study indicate a rapid positive influence of *S-*ketamine, primarily on anxiety. Patients who suffer from severe psychological distress may benefit from the positive effects of *S-*ketamine. The results are consistent with existing data related to ketamine and its effect on psychological distress. The rapid onset of *S-*ketamine action, as well as its anxiolytic and possible anti-depressant effects, can significantly improve the palliative care of patients. This study is limited due to non-randomization, retrospective design, and low sample size. Thus, there is a need for further studies.

## Supplementary information

**Additional file 1: Table S1.** Sample characteristics of the S-ketamine group and the control group.

**Additional file 2: Table S2.** Confounding variables.

**Additional file 3: Table S3.** Three-way mixed MANOVA; target variables: anxiety, depression; predictor variables: group, time and anxiety/depression (anxdep).

**Additional file 4: Table S4.** Two-way mixed ANOVA; target variables: anxiety and depression; predictor variables: group and time.

**Additional file 5: Table S5.** Two-way mixed MANCOVA (multivariate) and two-way mixed ANCOVA (univariate) with confounders as covariates.

**Additional file 6: Table S6.** Three-way mixed MANOVA (multivariate); three-way mixed ANOVA (univariate).

**Additional file 7: Figure S1.** Group means ± SD for STADI anxiety (A) and depression (B) values at T1 and T2.

## Data Availability

The datasets used and analyzed during the current study are available from the corresponding author on reasonable request.

## Abbreviations

A T1: Antidepressants at T1
AEDL: Activities and existential experiences of life
ANCOVA: Univariate analysis of covariance
ANOVA: Univariate analysis of variance
Anxdep: Anxiety vs. depression
B T1: Benzodiazepines at T1
FDA: The United States Food and drug administration
MANCOVA: Multivariate analysis of covariance
MANOVA: Multivariate analysis of variance
NRS: Numerical rating scale (for pain)
PSBS: Palliative symptom burden score
SPCU: Specialized palliative care unit
STADI: State trait anxiety depression inventory
T: T-values as standard score
T1: day of the last STADI evaluation before *S-*ketamine (control group: day of the first STADI evaluation from the second day of the stay)
T2: day of the first STADI evaluation after *S-*ketamine (control group: day of the next STADI evaluation after T1)
Z1: PSBS evaluation on the morning before *S-*ketamine (control group: PSBS evaluation on the morning of T1)
Z2: PSBS evaluation on the morning after *S-*ketamine (control group: PSBS evaluation on the morning of the day after T1)
