# Supplementary material for: A rapid positive influence of S-ketamine on the anxiety of patients in palliative care: a retrospective pilot study
Source: BMC Palliat Care. 2020 Jan 3;19:1. doi: 10.1186/s12904-019-0499-1 (PMC6942257; doi:10.1186/s12904-019-0499-1)
Supplement: Supplementary file 1 — Additional file 1: Table S1. Sample characteristics of the S-ketamine group and the control group. [file 12904_2019_499_MOESM1_ESM.docx]

Table S1: Sample characteristics of the *S*-ketamine group and the control group.

| **Variables** | **Categories** | ***S*-ketamine group** | **Control group** |
| --- | --- | --- | --- |
| Group size ^a^ |  | 8 | 8 |
| Gender ^a^ | Female | 4 | 5 |
|  | Male | 4 | 3 |
| Age ^b^ |  | 52.13 ± 13.25 | 54.00 ± 13.07 |
| Diagnosis at admission ^a^ | Bladder cancer | 0 | 1 |
|  | Breast cancer | 0 | 2 |
|  | Cancer of unknown primary | 1 | 0 |
|  | Cervical cancer | 1 | 1 |
|  | Glioblastoma | 1 | 0 |
|  | Colorectal cancer | 1 | 0 |
|  | HIV | 1 | 0 |
|  | Liver cancer | 0 | 1 |
|  | Lung cancer | 2 | 1 |
|  | Ovarian cancer | 0 | 1 |
|  | Pancreatic cancer | 0 | 1 |
|  | Prostate cancer | 1 | 0 |
| Length of stay in SPCU (days) ^b^ |  | 14.63 ± 7.69 | 13.63 ± 2.97 |
| Mode of discharge ^a^ | Home | 0 | 3 |
|  | Hospice | 3 | 2 |
|  | Other clinic | 0 | 2 |
|  | Died on the ward | 5 | 1 |
| Points of measurement (days) ^b^ | T1 | 5.63 ± 2.88 | 2.75 ± 0.89 |
|  | T2 | 8.00 ± 3.70 | 7.00 ± 2.62 |
|  | Z1 | 5.88 ± 2.90 | 2.75 ± 0.89 |
|  | Z2 | 6.88 ± 2.90 | 3.75 ± 0.89 |
| STADI anxiety ^b^ | T1 | 68.88 ± 11.01 | 62.63 ± 8.05 |
|  | T2 | 55.63 ± 11.73 | 64.00 ± 11.78 |
| STADI depression ^b^ | T1 | 66.38 ± 10.88 | 62.00 ± 10.77 |
|  | T2 | 57.75 ± 12.75 | 62.00 ± 11.02 |
| STADI global ^b^ | T1 | 68.38 ± 8.80 | 63.75 ± 9.22 |
|  | T2 | 57.38 ± 11.87 | 64.25 ± 9.56 |
| Pain ^b^ | Z1 | 3.88 ± 1.64 | 3.63 ± 2.00 |
|  | Z2 | 3.50 ± 1.77 | 2.63 ± 1.19 |
| Restlessness/anxiety ^c^ | Z1 | 1.00 (1.00 – 1.75) | 1.00 (0 – 2.00) |
|  | Z2 | 1.00 (1.00 – 1.00) | 1.00 (0 – 2.00) |

^a^ = *n*

^b^ = *M* ± *SD*

^c^ = *Mdn* (*IQR*)
