# Supplementary material for: A rapid positive influence of S-ketamine on the anxiety of patients in palliative care: a retrospective pilot study
Source: BMC Palliat Care. 2020 Jan 3;19:1. doi: 10.1186/s12904-019-0499-1 (PMC6942257; doi:10.1186/s12904-019-0499-1)
Supplement: Supplementary file 2 — Additional file 2: Table S2. Confounding variables. [file 12904_2019_499_MOESM2_ESM.docx]

Table S2: Confounding variables

| **Confounder** | **Points of measurement** | ***S*-ketamine group** | | **Control group** | |
| --- | --- | --- | --- | --- | --- |
| Pain ^a^ | T1 | 4.00 ± 1.85 | | 3.63 ± 2.00 | |
|  | T2 | 3.50 ± 1.77 | | 2.88 ± 1.46 | |
| AEDL ^a^ | T1 | 14.50 ± 7.69 | | 8.50 ± 4.14 | |
|  | T2 | 15.88 ± 7.70 | | 10.13 ± 4.76 | |
| Psycho-oncological treatment (minutes) ^a^ | T1 | 53.75 ± 55.92 | | 61.38 ± 65.96 | |
|  | T2 | 68.75 ± 68.86 | | 116.38 ± 97.46 | |
| Days with antidepressants ^a^ | T1 | 2.38 ± 2.97 | | 1.50 ± 1.77 | |
|  | T2 | 3.88 ± 4.12 | | 3.25 ± 3.37 | |
| Palliative care treatment (days) ^a^ | T1 | 5.63 ± 2.88 | | 2.75 ± 0.89 | |
|  | T2 | 8.00 ± 3.70 | | 7.00 ± 2.62 | |
| Benzodiazepines ^b^ | T1 | yes | 6 | yes | 5 |
|  |  | no | 2 | no | 3 |
|  | T2 | yes | 6 | yes | 4 |
|  |  | no | 2 | no | 4 |
| Antidepressants ^b^ | T1 | yes | 6 | yes | 3 |
|  |  | no | 2 | no | 5 |
|  | T2 | yes | 6 | yes | 4 |
|  |  | no | 2 | no | 4 |
| Opioids ^b^ | T1 | yes | 8 | yes | 8 |
|  |  | no | 0 | no | 0 |
|  | T2 | yes | 8 | yes | 8 |
|  |  | no | 0 | no | 0 |

^a^ = *M* ± *SD*

^b^ = *n*
