# Supplementary material for: A rapid positive influence of S-ketamine on the anxiety of patients in palliative care: a retrospective pilot study
Source: BMC Palliat Care. 2020 Jan 3;19:1. doi: 10.1186/s12904-019-0499-1 (PMC6942257; doi:10.1186/s12904-019-0499-1)
Supplement: Supplementary file 3 — Additional file 3: Table S3. Three-way mixed MANOVA; target variables: anxiety, depression; predictor variables: group, time and anxiety/depression (anxdep). [file 12904_2019_499_MOESM3_ESM.docx]

Table S3: Three-way mixed MANOVA; target variables: anxiety, depression; predictor variables: group, time and anxiety/depression (anxdep).

|  |  | **Test statistics** | **Significance 2-tailed** | **Effect size** |
| --- | --- | --- | --- | --- |
| **STADI scales** | **Effect** | ***F*(1, 14)** | ***p*** | ***r*** |
| Anxiety and depression | Group | 0.01 | 0.91 | 0.03 |
|  | Anxdep | 0.10 | 0.76 | 0.08 |
|  | Group x anxdep | 0.06 | 0.82 | 0.07 |
|  | Time | 3.98 | **0.066^+^** | 0.47 |
|  | Group x time | 5.11 | **0.040*** | 0.52 |
|  | Anxdep x time | 0.43 | 0.52 | 0.17 |
|  | Group x anxdep x time | 1.46 | 0.25 | 0.31 |

* *p*: statistical significance *p* < 0.05

**^+^** trend to statistical significance: 0.05 < *p* < 0.10
