# Supplementary material for: A rapid positive influence of S-ketamine on the anxiety of patients in palliative care: a retrospective pilot study
Source: BMC Palliat Care. 2020 Jan 3;19:1. doi: 10.1186/s12904-019-0499-1 (PMC6942257; doi:10.1186/s12904-019-0499-1)
Supplement: Supplementary file 4 — Additional file 4: Table S4. Two-way mixed ANOVA; target variables: anxiety and depression; predictor variables: group and time. [file 12904_2019_499_MOESM4_ESM.docx]

Table S4: Two-way mixed ANOVA; target variables: anxiety and depression; predictor variables: group and time.

|  |  | **Test statistics** | **Significance 2-tailed** | **Effect size** |
| --- | --- | --- | --- | --- |
| **STADI scale** | **Effect** | ***F*(1, 14)** | ***p*** | ***r*** |
| Anxiety | Group | 0.05 | 0.83 | 0.06 |
|  | Time | 6.12 | **0.027*** | 0.55 |
|  | Group x time | 9.29 | **0.009*** | 0.63 |
| Depression | Group | < 0.01 | 1.00 | 0.00 |
|  | Time | 1.76 | 0.21 | 0.33 |
|  | Group x time | 1.76 | 0.21 | 0.33 |

* *p*: statistical significance *p* < 0.05
