# Supplementary material for: A rapid positive influence of S-ketamine on the anxiety of patients in palliative care: a retrospective pilot study
Source: BMC Palliat Care. 2020 Jan 3;19:1. doi: 10.1186/s12904-019-0499-1 (PMC6942257; doi:10.1186/s12904-019-0499-1)
Supplement: Supplementary file 5 — Additional file 5: Table S5. Two-way mixed MANCOVA (multivariate) and two-way mixed ANCOVA (univariate) with confounders as covariates. [file 12904_2019_499_MOESM5_ESM.docx]

Table S5: Two-way mixed MANCOVA (multivariate) and two-way mixed ANCOVA (univariate) with confounders as covariates.

|  |  | **Multivariate** | |  | **Univariate** | | | | | | |
| --- | --- | --- | --- | --- | --- | --- | --- | --- | --- | --- | --- |
|  |  | **Anxiety & depression** | |  | **Anxiety** | | |  | **Depression** | | |
| **Covariates at T1 and T2** |  | **Test statistics** | **Sig. 2-tailed** |  | **Test statistics** | **Sig. 2-tailed** | **Effect size** |  | **Test statistics** | **Sig. 2-tailed** | **Effect size** |
|  | **Effect** | ***F*(2, 12)** | ***p*** |  | ***F*(1, 13)** | ***p*** | ***r*** |  | ***F*(1, 13)** | ***p*** | ***r*** |
| Pain | Group | 0.05 | 0.95 |  | 0.06 | 0.82 | 0.07 |  | 0.00 | 0.95 | 0.00 |
|  | Time | 2.05 | 0.17 |  | 4.40 | **0.056^+^** | 0.50 |  | 1.50 | 0.24 | 0.32 |
|  | Group x time | 4.68 | **0.031*** |  | 9.18 | **0.010*** | 0.64 |  | 1.63 | 0.22 | 0.33 |
| AEDL | Group | 0.22 | 0.80 |  | 0.30 | 0.59 | 0.15 |  | 0.41 | 0.54 | 0.17 |
|  | Time | 2.01 | 0.18 |  | 4.24 | **0.060^+^** | 0.50 |  | 2.87 | 0.11 | 0.43 |
|  | Group x time | 4.62 | **0.032*** |  | 8.73 | **0.011*** | 0.63 |  | 1.69 | 0.22 | 0.34 |
| Psycho­oncological treatment (minutes) | Group | 0.01 | 0.99 |  | 0.01 | 0.92 | 0.03 |  | 0.00 | 0.96 | 0.00 |
|  | Time | 4.75 | **0.030*** |  | 10.00 | **0.007*** | 0.66 |  | 4.85 | **0.046*** | 0.52 |
|  | Group x time | 3.45 | **0.065^+^** |  | 6.90 | **0.021*** | 0.59 |  | 0.69 | 0.42 | 0.22 |
| Days with anti-depressants | Group | 0.31 | 0.74 |  | 0.57 | 0.46 | 0.20 |  | 0.01 | 0.92 | 0.03 |
|  | Time | 1.05 | 0.38 |  | 2.02 | 0.18 | 0.37 |  | 0.29 | 0.60 | 0.15 |
|  | Group x time | 4.34 | **0.038*** |  | 8.81 | **0.011*** | 0.64 |  | 1.76 | 0.21 | 0.35 |
| Palliative Care Treat-ment (days) | Group | 0.16 | 0.86 |  | 0.33 | 0.58 | 0.16 |  | 0.03 | 0.86 | 0.05 |
|  | Time | 2.56 | 0.12 |  | 3.33 | **0.091^+^** | 0.45 |  | 5.36 | **0.038*** | 0.54 |
|  | Group x time | 3.63 | **0.058^+^** |  | 5.98 | **0.029*** | 0.56 |  | 0.30 | 0.60 | 0.15 |

* *p*: statistical significance *p* < 0.05

**^+^** trend to statistical significance: 0.05 < *p* < 0.10
