# Supplementary material for: A rapid positive influence of S-ketamine on the anxiety of patients in palliative care: a retrospective pilot study
Source: BMC Palliat Care. 2020 Jan 3;19:1. doi: 10.1186/s12904-019-0499-1 (PMC6942257; doi:10.1186/s12904-019-0499-1)
Supplement: Supplementary file 6 — Additional file 6: Table S6. Three-way mixed MANOVA (multivariate); three-way mixed ANOVA (univariate). [file 12904_2019_499_MOESM6_ESM.docx]

Table S6: Three-way mixed MANOVA (multivariate); three-way mixed ANOVA (univariate).

|  |  | **Multivariate** | |  | **Univariate** | | | | | | |
| --- | --- | --- | --- | --- | --- | --- | --- | --- | --- | --- | --- |
|  |  | **Anxiety & depression** | |  | **Anxiety** | | |  | **Depression** | | |
|  |  | **Test statistics** | **Sig. 2-tailed** |  | **Test statistics** | **Sig. 2-tailed** | **Effect size** |  | **Test statistics** | **Sig. 2-tailed** | **Effect size** |
| **Med** | **Effekt** | ***F(*2, 11)** | ***p*** |  | ***F(*1, 12)** | ***p*** | ***r*** |  | ***F*(1, 12)** | ***p*** | ***r*** |
| B T1 | Group | 0.11 | 0.90 |  | 0.08 | 0.78 | 0.08 |  | 0.24 | 0.63 | 0.14 |
|  | Time | 1.73 | 0.22 |  | 3.49 | **0.086^+^** | 0.47 |  | 0.89 | 0.36 | 0.26 |
|  | B T1 | 1.38 | 0.29 |  | 0.86 | 0.37 | 0.26 |  | 0.49 | 0.50 | 0.20 |
|  | Group x B T1 | 0.57 | 0.58 |  | 0.31 | 0.59 | 0.16 |  | 0.12 | 0.29 | 0.10 |
|  | Time x B T1 | 0.39 | 0.69 |  | 0.66 | 0.43 | 0.23 |  | 0.07 | 0.80 | 0.08 |
|  | Group x time | 4.27 | **0.042*** |  | 6.78 | **0.023*** | 0.60 |  | 0.52 | 0.48 | 0.20 |
|  | Group x time x B T1 | 1.42 | 0.28 |  | 0.02 | 0.89 | 0.04 |  | 1.29 | 0.28 | 0.31 |
| A T1 | Group | 0.30 | 0.75 |  | 0.65 | 0.44 | 0.23 |  | 0.09 | 0.78 | 0.09 |
|  | Time | 1.86 | 0.20 |  | 4.02 | **0.068^+^** | 0.50 |  | 2.17 | 0.17 | 0.39 |
|  | A T1 | 1.32 | 0.31 |  | 2.86 | 0.12 | 0.44 |  | 0.80 | 0.39 | 0.25 |
|  | Group x A T1 | 0.05 | 0.95 |  | 0.10 | 0.76 | 0.09 |  | 0.07 | 0.80 | 0.08 |
|  | Time x A T1 | 0.61 | 0.56 |  | 1.32 | 0.27 | 0.31 |  | 0.52 | 0.48 | 0.20 |
|  | Group x time | 2.52 | 0.13 |  | 5.13 | **0.043*** | 0.55 |  | 1.10 | 0.32 | 0.29 |
|  | Group x time x A T1 | 0.96 | 0.41 |  | 0.02 | 0.88 | 0.04 |  | 0.96 | 0.35 | 0.27 |

Med: Medication

B T1: Benzodiazepines at T1

A T1: Antidepressants at T1

* *p*: statistical significance *p* < 0.05

**^+^** trend to statistical significance: 0.05 < *p* < 0.10
