# Supplementary material for: A rapid positive influence of S-ketamine on the anxiety of patients in palliative care: a retrospective pilot study
Source: BMC Palliat Care. 2020 Jan 3;19:1. doi: 10.1186/s12904-019-0499-1 (PMC6942257; doi:10.1186/s12904-019-0499-1)
Supplement: Supplementary file 7 — Additional file 7: Figure S1. Group means ± SD for STADI anxiety (A) and depression (B) values at T1 and T2. [file 12904_2019_499_MOESM7_ESM.docx]

**Figure S1**

| 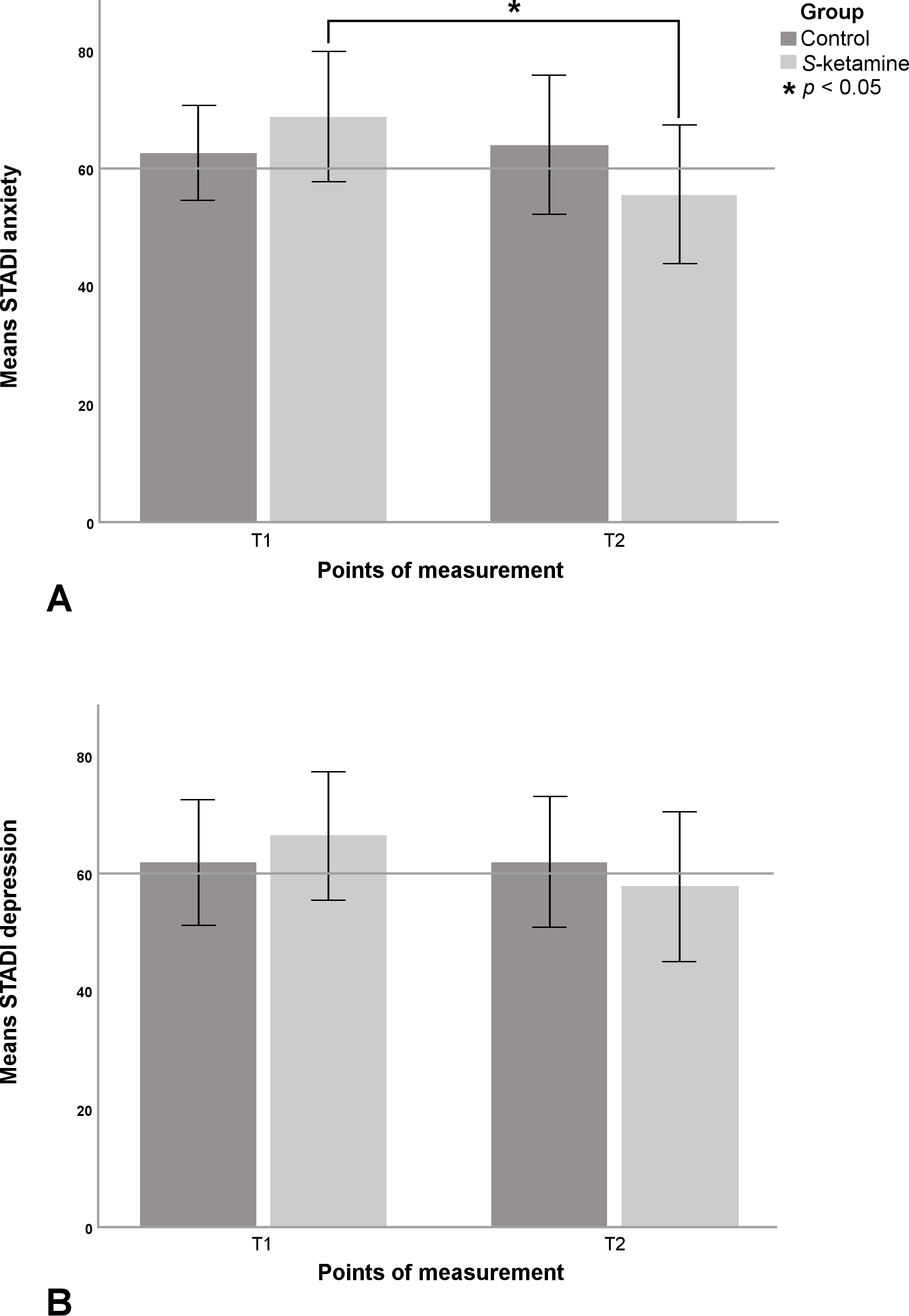 |
| --- |
| **Figure S1:** **Group means ± SD for STADI anxiety (A) and depression (B) values at T1 and T2.**  * *p*: statistical significance *p* < 0.05 of mean differences. |
